# Meta-analysis of the survival rate and postoperative infection rate of primary and secondary implants after vascularized fibula transplantation for reconstruction of jaw defects

**DOI:** 10.1186/s40729-023-00514-x

**Published:** 2023-12-18

**Authors:** Yi-bo Liu, Di Wu, Jun-yi Wang, Xiao-han Lun, Wei Dai

**Affiliations:** 1 Department of Oral and Maxillofacial-Head and Neck Surgery, School of Stomatology, Tianjin Medical University, Tianjin, China; 2 Department of Oral and Maxillofacial-Head and Neck Surgery, School of Stomatology, China Medical University, Nanjing North Street No.117, Shenyang, 110000 Liaoning China

**Keywords:** Mandibula reconstruction, Mandibula, Vascularized fibula flap, Primary implantation, Secondary implantation, Radiotherapy

## Abstract

**Objectives:**

Vascularized fibula flap transplantation is the most effective and common method to repair the jaw defects. In addition, implantation is the first choice to restore dentition on the graft fibula. Implants are usually implanted at least 6 months after fibula transplantation. Primary implantation of implants during surgery can restore the dentition earlier, but whether this method can achieve the same restorative effect as secondary implantation is still uncertain. This article aims to compare the survival rate and complications between primary and secondary implantation through meta-analysis.

**Methods:**

This meta-analysis was conducted according to PRISMA protocol and the Cochrane Handbook of Systematic Reviews of Interventions. According to the inclusion and exclusion criteria, we selected the PubMed, Embase, Web of Science, Cochrane Library, Chinese National Knowledge Infrastructure (CNKI), Chinese BioMedical Literature Database (CBM) according to established inclusion and exclusion criteria. The Newcastle–Ottawa Scale (NOS) was used to assess the quality of the included studies. Meta-analysis was conducted to compare the survival rate and postoperative infection rate of primary and secondary implantation.

**Results:**

Seven studies were involved in our research, involving 186 patients. Five of the studies detailed implant success in 106 patients (primary implantation 50, secondary implantation 56), and four studies documented infection after implantation in 117 patients (primary implantation 52, secondary implantation 65); the survival rate of the primary implantation was 93.3%, and the incidence of postoperative infection was 17.3%. The survival rate of the secondary implantation was 93.4%, and 23.1% had postoperative infection. Meta-analysis showed that there was no significant difference in the survival rate between primary implantation and secondary implantation, OR = 0.813 (95% CI 0.383–1.725, *P* = 0.589 > 0.05), and there was no significant difference in the incidence of postoperative infection, OR = 0.614 (95% CI 0.239–1.581, *P* = 0.312 > 0.05).

**Conclusions:**

Based on the results of this study, the research found no significant difference in the survival rate or infection rates between primary and secondary implantation. After appropriate indications selection, primary implantation can be used to reconstruct the dentition with less waiting time, reduce the impact of radiotherapy, and bring a higher quality of life for patients.

## Introduction

In the daily work of oral and maxillofacial surgery, developmental deformities, tumor resection, trauma and other reasons may cause jaw defects, which have a great impact on the appearance and function of patients. The use of autogenous bone transplantation to restore maxillofacial function is the mainstream repair method now, scapula, rib, radius, iliac crest, fibula and so on have been used for jaw repair. Among them, fibula transplantation was first proposed by Taylor in 1975, and was soon used in maxillofacial repair. In 1989, Hidalgo and colleagues reported the results of mandibular reconstruction using vascularized fibula flap in 13 patients [1, 2].Fibula has sufficient bone mass, enough blood supply, convenient to shape, less complications in donor site, strong anti-infection ability and lower long-term bone resorption than natural bone [3, 4]. These advantages make the vascularized fibula free flap a popular method for maxillofacial bone repair. The fibula graft restored the continuity of the mandible and recovered the patient's normal appearance. However, it is a tricky problem to restore the dentition of patients after fibula flap reconstruction.

With the development of implant technology, doctors tried to implant implants into the vascularized fibula to reconstruct the dentition and restore the occlusal function. For patients with benign tumors, implants are usually implanted 6 months after the surgery. On one hand, delayed implantation can avoid influencing the blood supply recovery of fibula; on the other hand, some studies believe that early implanted implants has poor osseointegration and lower success rate [5, 6]. There are also some disadvantages to secondary implantation, including longer waiting times for repairs and need a second operation. For patients who require adjuvant radiotherapy, implant placement takes longer, often 6–12 months after radiotherapy, which further prolongs the treatment time, and although implant repair is performed 12 months after radiotherapy, there is also a higher probability of radiation osteomyelitis after implantation [7, 8].

Because of the dental restoration need second operation and the long interval between the two operations, few patients successfully complete implant restoration after fibular repair. With the progress of technology, primary implantation has gradually attracted people's attention, which significantly reduces the repair time. Studies have proved that the primary implantation have a stable success rate after adjuvant radiotherapy [9]. However, whether primary implantation has similar implant success rate as secondary implantation is still inconclusive [10]. Some studies have compared the effects of the two methods, but the amount of data are small and the results are not completely consistent. This article aims to compare the survival rate and complications between primary and secondary implantation through meta-analysis, hoping to provide reference for clinical treatment by comparing the restoration effects of the two methods.

## Materials and methods

This meta-analysis complies with PRISMA and PICO guidelines, and its preparation followed criteria of the Cochrane Handbook for Systematic Reviews of Interventions.

### Search strategy

Two members searched literature in PubMed, Web of Science, EMBASE, Cochrane Library, Chinese National Knowledge Infrastructure (CNKI), and Chinese BioMedical Literature Database (CBM) published before October 2022, The English key is (mandible OR mandibular OR mandibles OR oral OR jaw OR maxillary) AND (fibula OR fibulas) AND (implant OR implants). The reference lists of relevant literature are searched to minimize omissions. The inclusion of controversial articles was evaluated and discussed by a third member until a consensus reached. Extracted the basic information of patients and other relevant data, including general information of patients, fibula implant survival rate, peri-implant bone resorption, peri-implant inflammation, complications, etc. Used endnote software to sort out the literature, and excluded the duplicate references.

### Inclusion and exclusion criteria

#### Inclusion criteria

1. Articles of randomised controlled trials or non-randomised controlled trials comparing primary and secondary implant methods after vascularized fibula flap transplantation.
2. Studies containing at least one of the following data: implant survival rate, bone resorption around the implant, peri-implantitis, incidence of postoperative complications, etc.

(Notes: In this study, because the data are not detailed enough, implant survival was defined as the total number of implants minus the number of lost or failed implants.)

#### Exclusion criteria

1. No data can be extracted from the research or cannot be used after contacting the author.
2. Meta-analyses, reviews, letters, conference abstracts, case reports and editorials were excluded.

The Newcastle–Ottawa scale (NOS) was used to evaluate the quality of the included literature. When the score was greater than or equal to 5, the quality of the study was considered good. Statistical analysis of meta-analyses was performed using STATA11.0 software, low heterogeneity between studies was considered when *P* > 0.1, *i*^2^ < 50% was tested for heterogeneity, using a fixed-effects model; when *I*^2^ > 50%, the heterogeneity among studies was considered to be high, and the random effects model was used. There was statistical significance when *P* < 0.05. Sensitivity analysis was used to assess the stability of the statistical results, and Egger’s test was used to assess publication bias between articles.

## Results

**Fig. 1:**
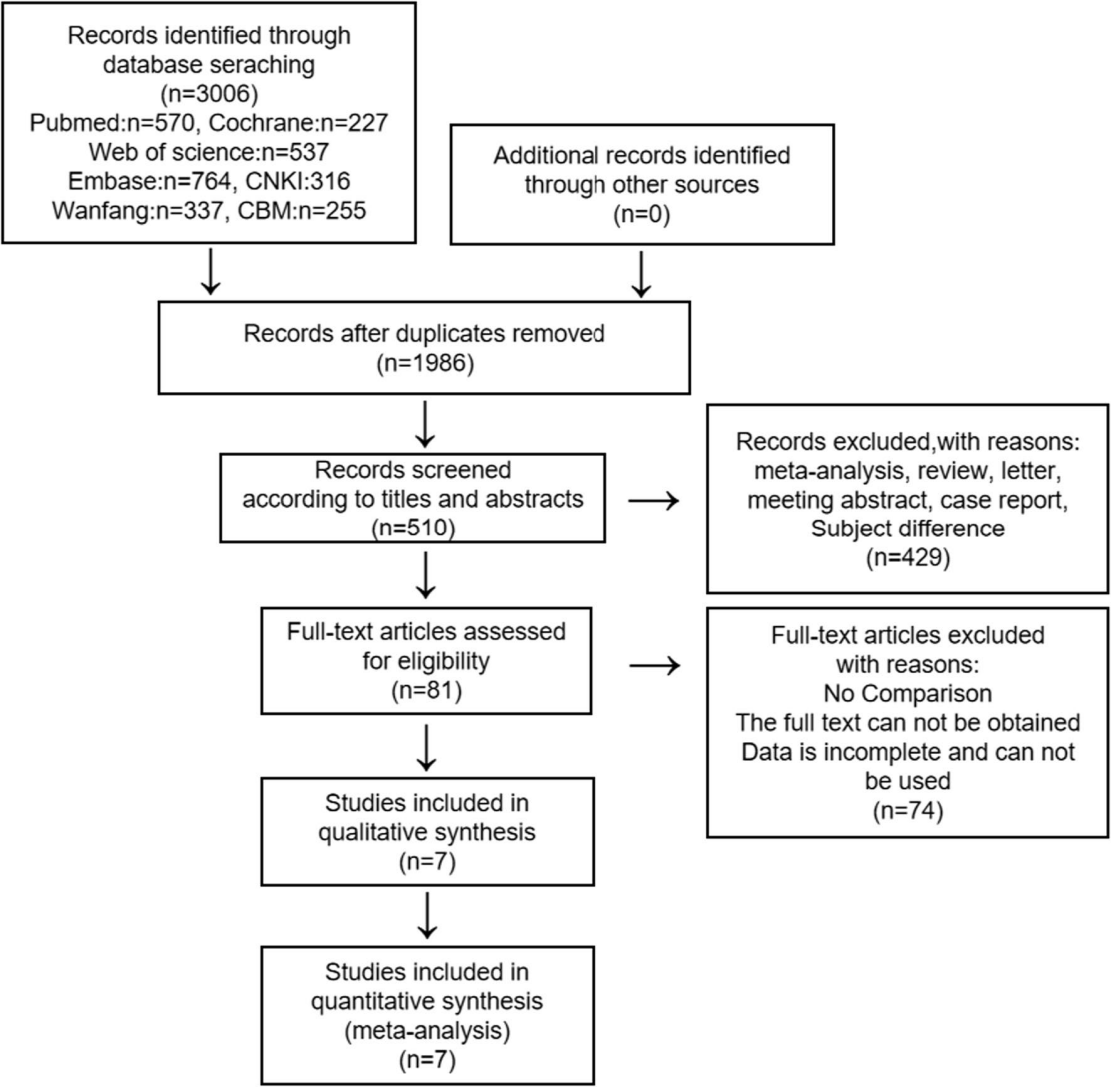
PRISMA flow chart of literature screening

After searching the database and removing the duplicates, we got 1986 articles, 510 were related to fibula implantation. After further screened, we removed the non-comparative articles and articles with incomplete data, selected 7 articles [9, 11–16], all of these articles published from 1997 to 2020, 5 articles were published after 2015, all articles were retrospective studies. The article screening process is shown in Fig. 1.

Table 1 assessed the quality of the literature, the full mark of the selection is 4, comparability is 2, and exposure is 3, when the score was greater than or equal to 5, the quality of the study was considered good. The overall quality of the literature is good, the literature sorted out the basic data (Table 2). A total of 186 patients from seven articles were enrolled in the study. Most of the patients were between 45 and 65 years, the average age was about 58.3 years. 23 patients were diagnosed with benign tumors and 114 with malignant tumors, 39 with radiation osteomyelitis, 6 with osteomyelitis, and 4 with post-traumatic repair. All patients were treated with vascularized fibula flap transplantation and implantation in fibula repair area. 5 out of 7 literatures included the postoperative effects of simultaneous implantation and delayed implantation [9, 13–16], a total of 50 patients were implanted with 208 implants in their first surgery and 194 survived, the implant survival rate was 93.3%. 56 patients used secondary implantation method, who were implanted with 229 implants and 214 survived, the implant survival rate was 93.4%. The postoperative complications associated with implantation include granulation tissue hyperplasia, bone exposure or poor union, local infection, wound dehiscence, bone fracture, etc. The highest incidence was local infection, there were 4 articles about the infection after fibula implantation [11, 12, 14, 16], including fistulas, local cellulitis. 52 patients were implanted in the first stage, the infection rate was 17.3%, 65 patients were implanted in the second stage, postoperative infections occurred in 23.1% of cases, including fistulas and local cellulitis.

**Table 1:** Literature quality evaluation

Author	Selection	Comparability	Exposure	Score
Ryan S. Jackson	★★★★	★	★★★	8
María L. Sandoval	★★★	★	★★	6
Robert J. Allen,	★★★★	★	★★	7
Deanna C. Menapace	★★★★	★	★★★	8
Eleni D. Roumanas	★★★	★	★★★	7
Bernardo Bianchi	★★★★	★	★★★	8
Fatih Cabbar	★★★★	★	★★★	8

Each ★ represents a point

**Table 2:** Basic information of literature was included

Author	Years	Period	Patient	Male/female	Age	Implant	Fail	Malignant tumour	Benign tumour	Other diagnosis	Infection	Radiotherapy history	Follow-up period
Ryan S. Jackson	2016	Primary	20	13/7	53.9 (16.6–79.0)	80	4	9	3	8	–	11	Mean 22 months
		Secondary	26	18/8	61.2 (25.3–80.3)	103	8	17	1	8	–	16	
María L. Sandoval	2019	Primary	10	8/2	70 (50–74)	–	–	10	0	0	2	3	1 year
		Secondary	10	7/3	55 (40–73)	–	–	10	0	0	3	2	
Robert J. Allen	2020	Primary	27	21/6	63.07 ± 8.18	72	1	24	0	3	3	15	3 months
		Secondary	33	24/10	56.06 ± 15.86	–	–	30	0	3	6	21	
Deanna C. Menapace	2018	Primary	12	8/4	59.8 (24–79)	49	6	–	–	12	4	–	20 months
		Secondary	11	8/3	65.0 (59–81)	52	5	–	–	11	5	–	31 months
Eleni D. Roumanas	1997	Primary	3	1/2	–	13	2	2	0	1	0	2	–
		Secondary	11	6/6		44	2	10	0	1	1	5	49 months
Bernardo Bianchi	2013	Primary	11	6/5	47.36 ± 14.36	52	0	0	11	0	–	0	mean 53.6 months
		Secondary	4	3/1	58.5 ± 8.5	18	0	0	4	0	–	0	
Fatih Cabbar	2018	Primary	4	4/4	46.8 ± 13.2	14	2	2	4	2	–	1	**–**
		Secondary	4			12	0				–		–

**Fig. 2:**
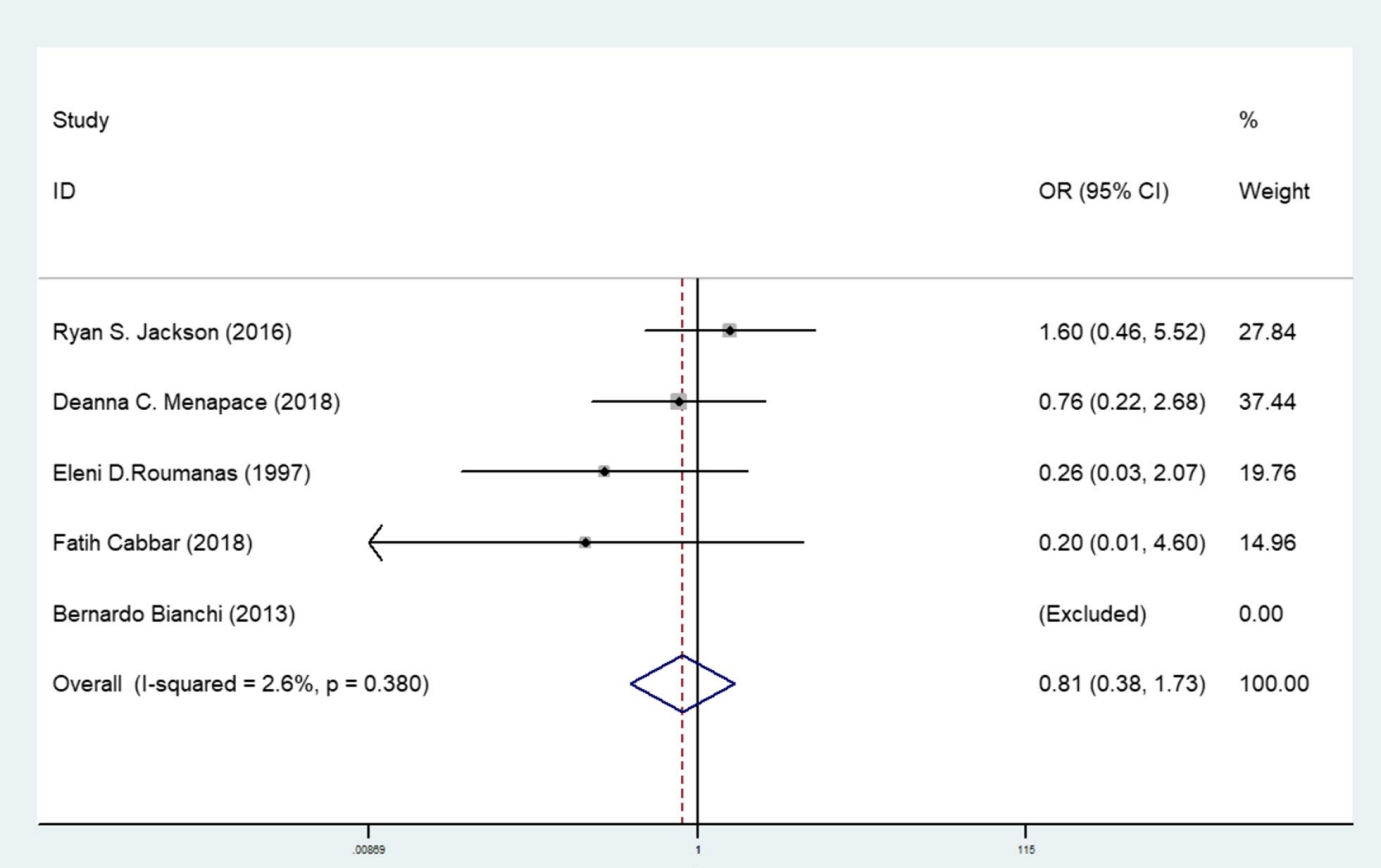
Forest plot of the survival rate comparation

We used Stata11.0 to analyse the survival rate of primary and secondary implantation in 5 articles, compared the effects of the two methods using a fixed-effects model. First, the heterogeneity test was performed, *P* = 0.380, *i*^2^ = 2.6%, so the fixed-effects model was used for analysis. The meta-analyse results indicated there was no significant difference in the survival rate between two groups, OR = 0.813 (95% CI 0.383–1.725, *P* = 0.589), *P* > 0.05, indicating that there was no significant difference in implant survival rate between the primary and secondary implantation (Fig. 2).

**Fig. 3:**
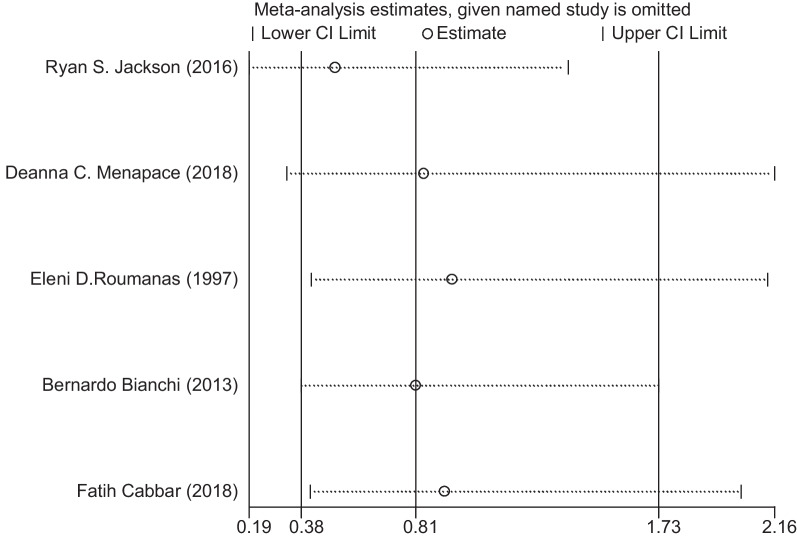
Sensitivity analysis of the survival rate comparation

**Fig. 4:**
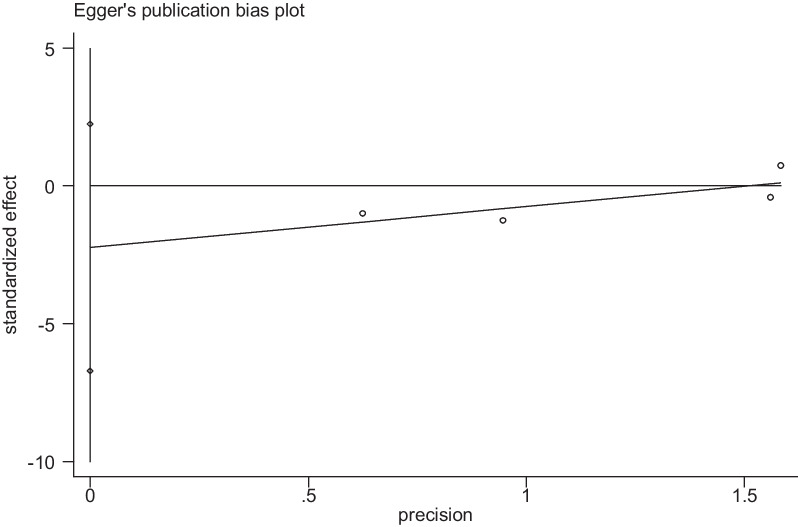
Egger's test of the survival rate comparation

At the same time, we analyse the sensitivity analysis and publication bias analysis. In the sensitivity analysis, when we excluded individual articles, there are no significant change in the statistical results, demonstrating that our results were not affected by any single study (Fig. 3). Egger’s test results *P* = 0.165 > 0.05 showed no apparent publication bias. These tests indicated low heterogeneity across articles and robust results (Fig. 4).

**Fig. 5:**
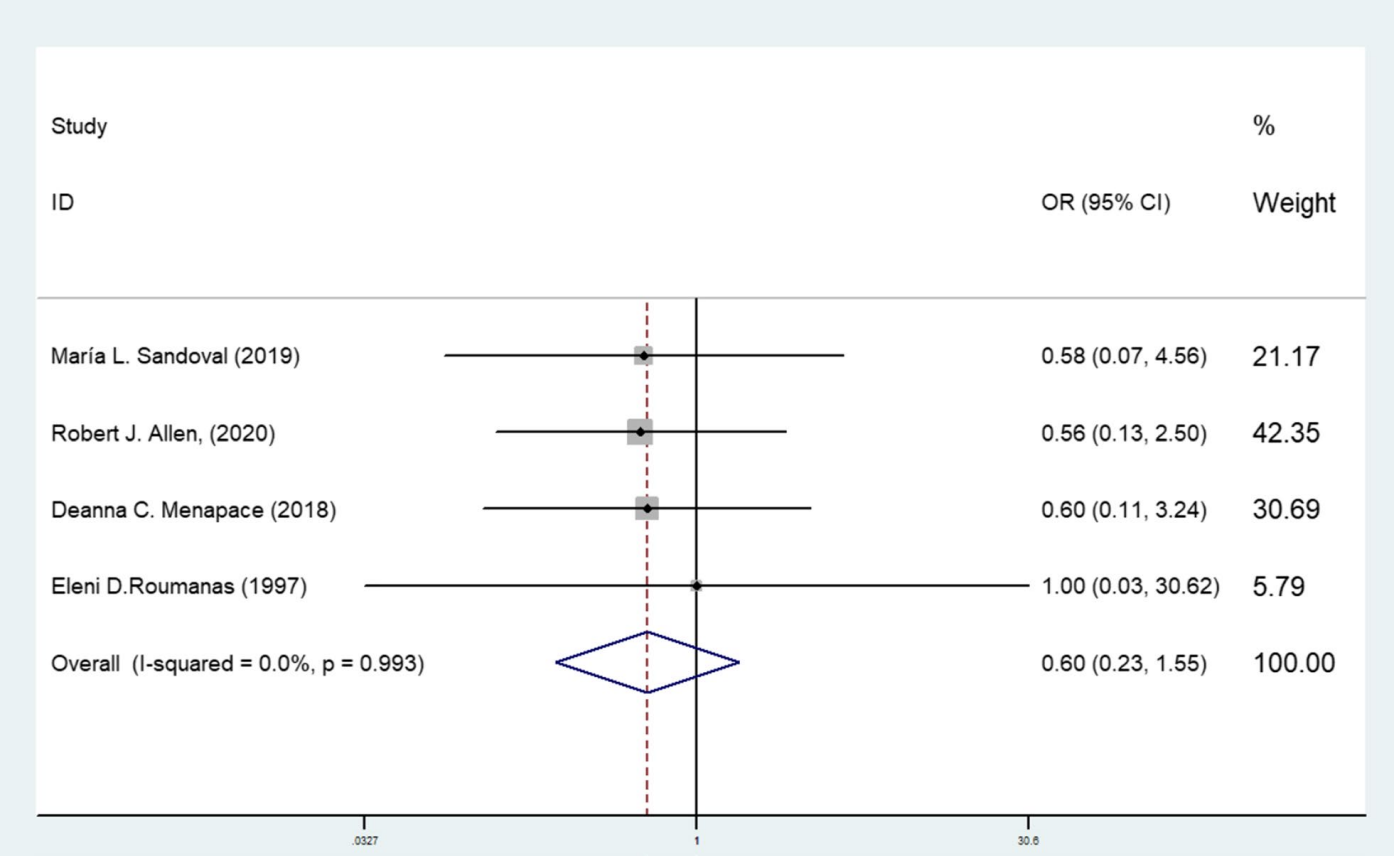
Forest plot of the postoperative infection comparation

We also analyzed the local postoperative infection in 4 articles, The results of heterogeneity test were *P* = 0.993, *i*^2^ = 0%, and were analyzed by fixed-effects model. The results showed that there was no significant differences in the incidence of postoperative infection between two methods, OR = 0.604 (95% CI 0.235–1.553, *P* = 0.295 > 0.05) (Fig. 5).

**Fig. 6:**
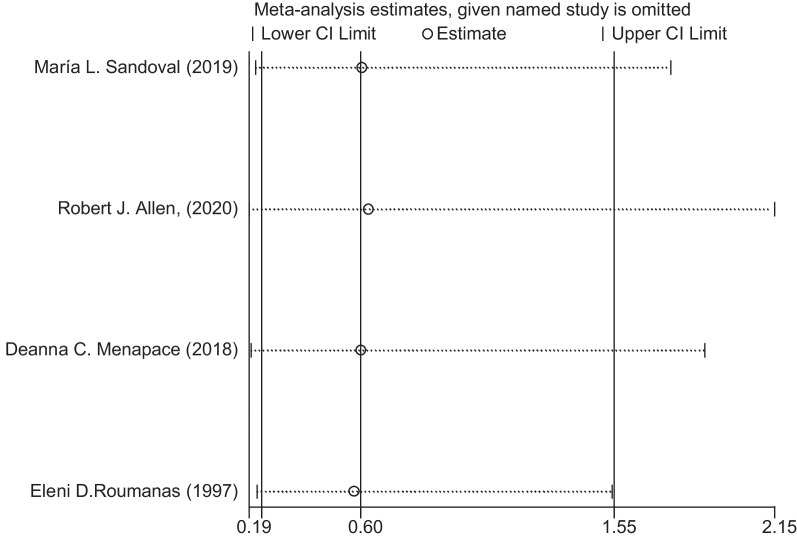
Sensitivity analysis of the postoperative infection comparation

**Fig. 7:**
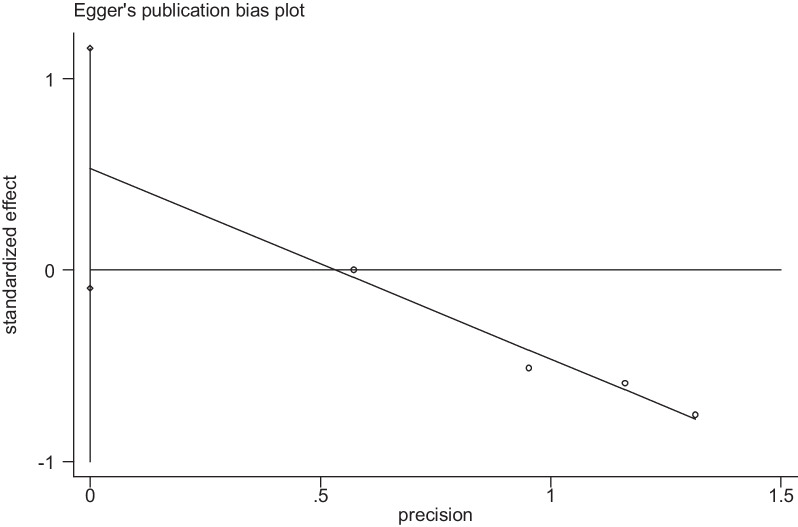
Egger's test of the postoperative infection comparation

For the sensitivity analysis and publication bias analysis, in the sensitivity analysis, when we excluded individual articles, there are no significant change in the statistical results, demonstrating that our results were not affected by any single study (Fig. 6). Egger’s test results *P* = 0.068 > 0.05 showed no apparent publication bias (Fig. 7). These tests indicated low heterogeneity across articles and robust results.

With these two statistical results, we can conclude that for patients after vascularized fibula flap transplantation reconstructed, the use of primary and secondary implantation method can get similar survival rate and postoperative infection rate.

## Discussion

With the increasing use of primary implantation, many researchers were comparing primary implantation with secondary implantation, hoped to find more effective restoration method. However, because of a series of reasons, such as ethical issues, the number of relevant studies was small, most of them only had small sample size, and these researches have different results, cannot reflect the results of this question. The conflict of these results also makes it difficult to choose the two methods in clinical practice. This meta-analysis compares the effect of primary implantation and secondary implantation after fibula flap transplantation by summarizing several articles comparing the two methods. According to our results, there was no significant differences in the survival rate and complication rate between primary implantation and secondary implantation, which provided the basis for using primary implantation to restore dentition, it is suggested that the effect of simultaneous implantation is reliable and can be used in clinical reconstruction for patients with appropriate indications.

Compared with the secondary implantation, the primary implantation only needs one operation, which not only reduces the patient’s pain, saves the treatment cost, but also greatly reduces the patient’s recovery process, dentures can be installed in 3–6 months after fibula surgery, which is important for function and aesthetics, helps patients return to normal life as soon as possible [17]. Especially for the patients who need adjuvant radiotherapy, the patients with primary implant can carry out radiotherapy on time without affecting the restoration time of the dentition and the bone union of the implant, because at that time, the osseointegration of the implants is almost complete [9].

Hina Panchal’s article included a larger sample of cases, Among them, 269 implants were implanted in 60 patients, the success rate is 97%, 1897 implants were implanted in 597 patients, the success rate is 89.9%), concluded that the success rate of primary implantation was higher than secondary implantation [18], which is different from our results. We consider Hina Panchal’s study is a systematic review, the included literature was not limited to comparative articles, many articles included the results of ilium implants, has more heterogeneous, and the follow-up time for the two procedures was not equivalent (14 vs 40 months), these reasons may influenced the study results [18]. Alan Ardisson’s systematic review also noted that the success rates of primary and secondary implantation were similar (95%, 93–100% for the same period; 91%, 83–100% for the second period) [19], but the data were not rigorous enough, at the same time, there is no in-depth discussion on how to select the two methods. Due to the insufficient number of related articles, the amount of literature included in this paper is relatively limited, but the homogeneity and comparability are good, further completing the quantitative analysis from the qualitative perspective, the results are more reliable and can be used as the basis for the selection of clinicians.

Retrospective studies may present incomplete data and unclear descriptions. To incorporate accurate data to obtain more realistic results, we carefully read the full text of each article in the data statistics in this article, removing data that might have contributed to bias, like cases of implant loss due to reoperation because of fibula necrosis or tumor recurrence are excluded, data bias is minimized by this approach. However, some biases cannot be excluded, for example, in the case of secondary implantation, to ensure the success of the repair operation, the doctor will evaluate the local recovery of the patient first and then decide whether to proceed with a secondary implantation operation. For patients with poor local recovery after implantation, it may be possible to extend the waiting time to observe local recovery, or to perform targeted treatments to promote local environmental compliance with implantation criteria. However, for primary implantation, the physician cannot predict the patient's future recovery before surgery, the choice of primary implantation can only be made according to the preoperative and intraoperative conditions, which makes the survival rate of primary implantation have a natural disadvantage compared with secondary implantation, and cannot be ruled out. In addition, although the success rate of vascularized fibula transplantation has been very high, it still has a failure rate of about 2–5%. The most common cause of fibula failure is vascular crisis, the majority of cases occur within a week after fibula transplantation, and the long-term incidence of fibula necrosis is low [1]. For primary implants, failure in fibula is equivalent to failure of the implant, but the fibula failure rate need not be considered in the statistics of secondary implant, which also reduces the survival rate of the primary implantation.

In clinical practice, the commonly used criteria for implant success are the standard from Albrektsson and Zarb in 1986 and from the Chinese Journal of Stomatology in 1995 [20]. In this study, because of the different publication times of the included articles, the criteria for implant success were also different, and some of the articles were followed for less than 3 years. Therefore, it is not possible to compare the implant success rate under the same condition. In addition, the total number of implants that fell off or failed due to infection or poor bone union were not clearly recorded in all the literature, so in this study, implant survival was defined as the total number of implants minus the number of lost or failed implants. This calculation method may not be able to reflect the status of retained implants good, with some conditions neglected, such as implant mobility, the surrounding inflammation, but it is still a good representative of the postoperative effect of implants.

In the literature included in this study, all dental implants were completed and functional, but the method of dental restoration was not accurately described in most of the literature. The long-term survival rate of dental implants will be different with different dentition restoration methods. Therefore, failure to categorize the types of dental restorations in each article may increase the bias in the result. However, the literature included in this study was all comparative, in a single article, it have compared primary implantation with secondary implantation and come to a conclusion, so we can assume that in the same article, other factors had little influence on our statistical results, such as the way of dentition restoration.

Although the success rate of primary implantation has now reached an ideal level, it still faces some difficulties. In secondary implantation, the operator can retake the CT before implant operation and design it to ensure the accuracy of implant location. However, for primary implantation, it is difficult to place the implant in predetermined position accurately during the surgery, even we used CAD/CAM technology to design and print the guide plate before operation, the angle between the fibula and the mandible may deviate when the fibula in place or when the implant placed in the fibula, these small deviations can add up to poor implant angles, which can affect the effectiveness of implant restoration, even lead to the final implant cannot load the crown [17, 21, 22]. There are many ways to improve the accuracy of prosthetics, such as extending the implant guide plate to the jaw to increase retention a when printing the guide plate in 3D print [23], use occlusal splint to adjust the placement of the implant, designing the implant guide plate and fibular osteotomy guide plate as a whole to reduce the error of removing the guide plate, or implanting the implant into the fibula before transfer the flap into the mouth, these allow the implant implanted in an ideal location [24–26]. As an emerging technology, surgical navigation technology can make the surgical process visible and further improve the accuracy of surgery [27–29].

Six of the seven articles included in this meta-analysis included patients who had undergone radiotherapy [9, 11–16], there was no consensus that radiotherapy would reduce the success rate of intraosseous implants, but many researchers are conservative about fibula implants after radiation therapy, and some studies have confirmed this concern [30–32], it is considered that radiotherapy is one of the risk factors affecting the survival rate of implants. To be on the safe side, we should wait at least 12 months for a recovery period before attempting an implant, and implant in 12–24 months, it is also convenient to observe the recovery of the local operative area [7, 30]. Some studies suggest that the risk of implant failure is significantly increased in 12 months after radiotherapy, the bone regenerative ability was inhibited by 70.9% after radiotherapy and recovered by 28.9% within 1 year [30]. Waiting times should not be too long, Granstrom said, implant success rates may be lower after too long wait time (> 10 years), because progressive arterial endocarditis affects the ability of bone to heal. However, there is a short-term positive cellular effect after radiotherapy that can improve bone healing [28]. After 2–3 years of implants survival, the long-term success rate is not related to radiotherapy, but more dependent on the environment around the implants. Some research appears that dental implantation also does not affect FFTT survival in patients with a history of osteoradionecrosis and osteonecrosis [13]. In the past, patients who had adjuvant radiotherapy were often advised to undergo Hyperbaric Medicine before implant surgery, because it promoted angiogenesis and offset some of the radiation damage, but for implant therapy, the Hyperbaric medicine did not significantly improve implant success [33, 34].

In contrast, primary implantation may be a better way to avoid the potential risks of radiation therapy, some studies suggest that radiotherapy does not affect the osseointegration of implants [9, 31, 35, 36]. María believe that the presence of implants does not increase the risk of complications after surgery or during radiation therapy [11], nor does it affect the success rate of implants in patients with previous radiation necrosis [8, 11]. Navarro considered that bone integration was almost complete after 3–4 months, during this time radiotherapy had not affected bone vessel formation [9, 13, 37]. Hina’s systematic review showed that although there is no statistically significant, implants placed before radiotherapy had a higher survival rate than after radiotherapy (*P* = 0.07) [18].

Many of the patients included in this study had been treated with radiotherapy before, and the radiotherapy of each patient was not clearly described in the literature, so an effect of radiotherapy on the results of this study could not be excluded. In the above discussion, although the negative effects of radiotherapy on implants are not clear, many findings suggest that concurrent implantation before radiotherapy can reduce the potential risks of implantation. If we excluded the effect of radiation therapy, the survival rate of delayed implantation may be slightly improved; however, whether the change of implant survival rate caused by radiotherapy will affect our final results cannot be determined only by the present evidence.

In terms of operating time, primary implantation prolongs the operative time at least 40 min, potentially increasing the risk of postoperative systemic complications. However, compared with secondary surgery, we consider that it is worthwhile to extend some operation time. The failure rate of vascularized fibula is a significant risk for primary implantation, if there is a flap vascular crisis and cannot be rescued, the soft tissue can be removed, change to non-vascularized fibula flap, but we should close the wound more carefully to seal the wound, and the incidence of postoperative local inflammation is also greatly increased [38]. In addition, some studies have suggested that primary implants may not appropriate for short bone grafts, because it will impact the recovery of local blood supply [11].

The selection of planting time is based on the specific situation. According to our results, for patients with non-malignant tumors, if patients and their families have the willingness to implant repair, have good economic and physical conditions, we can carry out primary implantation. For malignant tumor, if the tumor scope is small, the malignant degree is low, and the operation process is great, if the patients and their family members are more willing to perform implant repair, we can consider implanting implants at the primary surgery, for patients who require adjuvant radiation therapy, the doctor may recommend the primary implantation to reduce the potential risk of radiation therapy. For patients with higher malignancy and larger resection range, it is more important to follow up the surgical area, so secondary implantation may be more appropriate to avoid unnecessary treatment. However, the early restoration of occlusal function has a positive regulative effect on the patients’ diet and psychological state, so doctors should consider the patients’ own needs in clinical treatment, and combined with economic factors, patient’s condition and so on to choose a more suitable program.

There is also some deficiencies in this article. First of all, the 7 articles included in this paper are all retrospective studies, the total number of patients included is only 200 people, the sample size is not large enough, the results may have some errors, individual differences in patients and contingencies have a greater impact on overall outcomes, the consistency among cases, such as the follow-up time, the surrounding environment of the implant, the selection of the superstructure, cannot be completely consistent, it needs more prospective studies to verify. In addition, there are many kinds of complications in this article, the most common complication is local infection, the most common complication, was chosen to reflect the occurrence of the complication from one side. The results showed that there was no significant difference in the infection rate between the two kinds of operations.

## Conclusion

The survival rate of primary implantation and secondary implantation after vascularized fibula transplantation was similar, and there was no significant difference in the postoperative infection rate between two groups. The result suggested that primary dental implantation can be used as a reliable method in clinic to reduce the repair time and to restore dentition earlier. Of course, how to choose the two methods requires close cooperation between doctors and patients, to determine a most appropriate, both sides satisfactory program, and bring better treatment results for patients. At last, we hope more research about implant and radiotherapy of fibula in the future, and make the technique of jaw reconstruction more perfect and mature.

## Data Availability

The data sets used and/or analyzed during the current study are available from the corresponding author on reasonable request.

## Abbreviations

CAD/CAM: Computer-Aided Design and Manufacturing
CT: Computer Tomography
NOS: Newcastle–Ottawa Scale
